# Simvastatin improves the homing of BMSCs *via* the PI3K/AKT/miR‐9 pathway

**DOI:** 10.1111/jcmm.12795

**Published:** 2016-02-12

**Authors:** Weidong Bing, Xinyan Pang, Qingxi QU, Xiao Bai, Wenwen Yang, Yanwen Bi, Xiaolu Bi

**Affiliations:** ^1^ Department of Cardiovascular SurgeryQi Lu Hospital of Shandong UniversityJinanShandong ProvinceChina; ^2^ School of Life Science of Shandong UniversityJinanShandong ProvinceChina

**Keywords:** CXCR4, simvastatin, AKT, microRNA, BMSCs

## Abstract

Bone marrow‐derived mesenchymal stem cells (BMSCs) have great therapeutic potential for many diseases. However, the homing of BMSCs to injury sites remains a difficult problem. Recent evidence indicates that simvastatin stimulates AKT phosphorylation, and p‐AKT affects the expression of chemokine (CXC motif) receptor‐4 (CXCR4). Therefore, simvastatin may improve the expression of CXCR4 in BMSCs, and microRNAs (miRs) may participate in this process. In this study, we demonstrated that simvastatin increased both the total and the surface expression of CXCR4 in BMSCs. Stromal cell‐derived factor‐1α (SDF‑1α)‐induced migration of BMSCs was also enhanced by simvastatin, and this action was inhibited by AMD 3100(a chemokine receptor antagonist for CXCR4). The PI3K/AKT pathway was activated by simvastatin in this process, and LY294002 reversed the overexpression of CXCR4 caused by simvastatin. MiR‐9 directly targeted CXCR4 in rat BMSCs, and simvastatin decreased miR‐9 expression. P‐AKT affected the expression of miR‐9; as the phosphorylation of AKT increased, miR‐9 expression decreased. In addition, LY294002 increased miR‐9 expression. Taken together, our results indicated that simvastatin improved the migration of BMSCs *via* the PI3K/AKT pathway. MiR‐9 also participated in this process, and the phosphorylation of AKT affected miR‐9 expression, suggesting that simvastatin might have beneficial effects in stem cell therapy.

## Introduction

Bone marrow‐derived mesenchymal stem cells (BMSCs) have great therapeutic potential for many diseases, such as ischaemic heart disease, end‐stage liver disease, and ischaemic stroke 1, 2, 3, 4, 5. However, the homing of BMSCs to injury sites remains a difficult problem, impairing their clinical application. The stromal cell‐derived factor‐1α (SDF1‐1α)/chemokine (CXC motif) receptor‐4 (CXCR4) axis plays an important role in the homing of BMSCs 6. The up‐regulation of CXCR4 can improve the therapeutic effects of BMSCs 7, 8.

The PI3K‐AKT signalling pathway regulates the biological activities of cells, including the homing of BMSCs. Cartilage oligomeric matrix protein‐angiopoietin‐1 (COMP)‐angiopoietin 1 increases the migration of BMSCs *via* Tie‐2‐mediated activation of the PI3K/AKT signalling pathways 9. AKT activation promotes prostate tumour growth and metastasis *via* CXCL12/CXCR4 signalling 10.

Simvastatin, a 3‐hydroxy‐3‐methylglutaryl‐coenzyme A reductase inhibitor, decreases serum cholesterol. Other benefits of simvastatin include enhancing osteogenesis and endothelial differentiation of BMSCs 11, 12. If simvastatin influences the homing of BMSCs, it would advance the clinical administration of BMSCs. Simvastatin activates the PI3K‐AKT signalling pathway in endothelial progenitor cells (EPCs) 13, endothelial cells 14, and podocytes 15. Simvastatin may also play this role in BMSCs. As discussed above, the increased phosphorylation of AKT may lead to overexpression of CXCR4. In general, simvastatin may promote the homing of BMSCs. Yang and colleagues reported increased numbers of DAPI‐labelled cells in the hearts of pigs treated with simvastatin + BMSCs compared with pigs exclusively treated with BMSCs, and they attributed this increase to cell survival 16. It is possible that simvastatin enhances the homing of BMSCs; therefore, we determined whether simvastatin induces CXCR4 expression in BMSCs.

MicroRNAs participate in the post‐transcriptional regulation of mRNAs and modulate the biological features of cells 17. Various miRs regulate the expression of CXCR4. MiR‐150 targets CXCR4 in bone marrow‐derived mononuclear cells, and 18 miR‐494‐3p regulates CXCR4 expression in prostate cancer cells 19. We asked whether one or more miRs participate in the simvastatin‐regulated expression of CXCR4.

In our study, we assessed the effect of simvastatin on the expression of CXCR4 and determined whether the PI3k‐AKT pathway participates in this process. The expression of miRs that target CXCR4 was also examined.

## Materials and methods

The simvastatin prodrug was purchased from (Sigma‐Aldrich, St. Louis, MO, USA) and was subjected to activation as described by Sadeghi, *et al*. 20 Chloro‐methylbenzamido dialkylcarbocyanine (CM‐DiI) was purchased from Invitrogen (Carlsbad, CA, USA). The PI3K inhibitor, LY294002, was purchased from Abcam (Cambridge, MA, USA), and the SDF‐1α/CXCR4 cascade antagonist, AMD 3100, was obtained from Selleck Chemicals (Houston, TX, USA),. SDF‐1α was procured from Proteintech Group (Chicago, IL, USA). The inhibitor and mimic of miR‐9 were synthesised by Bio‐Asia (Jinan, China). The reporter constructs, pGL3‐CXCR4 and pGL3‐mutCXCR4, with a mutated target seed sequence of miR‐9, were also obtained from Bio‐Asia. Real‐time quantitative polymerase chain reaction (PCR) primers were designed by GeneCopia.

### Ethics

The present study was performed in accordance with the Declaration of Helsinki and the guidelines of the Ethical Committee of the Qilu Hospital of Shandong University (Jinan, China).

### Isolation, culture and labelling of BMSCs

The isolation and culture of BMSCs were performed as previously described 21. Briefly, BMSCs were extracted from the femurs of young Wistar rats (male, weighing 60–80 g) under anaesthesia with pentobarbital sodium (50 mg/kg). The culture medium was ɑ‐MEM with 10% (v/v) foetal bovine serum. The bone marrow was flushed and then cultured. Removal of haematopoietic cells was achieved by washing with phosphate‐buffered saline (PBS) and changing the culture medium at 48 h. Cells used in the experiments were from passages 3 to 5.

Before transplantation, cells were labelled with CM‐DiI according to the manufacturer's instruction.

### Vein grafting model

Vein grafting was performed similarly to our previous study with cuff technique and Left external jugular vein (LEJV) was autologously inserted to left common carotid artery (LCCA) 22. Adult male Wistar rats weighing 250 to 300 g were used in this experiment. 2‐mm body loops of cuffs were fetched from a 20‐GA intravenous cannula (BD, Sweden). LEJV was excised and washed, which was about 20‐mm long. Both ends of LEJV were turned inside out over the cuffs and ligated with 8‐0 silk to avoid slippage of two cuffs. After a longitudinal arteriotomy of LCCA, each cuff was inserted into the carotid artery and ligated to the artery wall. To permit extension of vein graft, back wall of LCCA between the cuffs was cut away. Occlusion of LCCA ranged from 5 to 10 min and blood flow restored immediately after the clamps were removed. Heparin (100 U in 0.2 ml) was given immediately before and after grafting. To observe the migration of BMSCs, fewer cells we used than our previous study 22. 0.2 ml CM‐DiI‐labelled cells (1 × 10^8^/ml) transplanted through caudal vein immediately after grafting. Sub‐therapeutic dose of Simvastatin (0.5 mg/kg) was gavaged daily for whole experiments according to Xu Cui and colleagues 23. Animals received AMD3100 5 mg/kg/day intraperitoneally for the whole experiments 24. Five groups were created after grafting: (1) Control group, *n* = 12, (2) Vein grafting group, *n* = 12, (3) MSC transplantation group (vein grafting with MSC transplantation), *n* = 24, (4) Simvastatin group (vein grafting with MSC transplantation and administration of Simvastatin), *n* = 24, (5) Simvastatin and AMD 3100 group (vein grafting with MSC transplantation and administration of Simvastatin and AMD3100), *n* = 24.

12 rats in each group except for the Vein grafting group or Control group were humanely killed at 7 days for evaluation of BMSCs distribution by observation of fluorescence in frozen serial sections of vein grafts. All the left rats were sacrifice 4 weeks after the operation and HE staining of paraffin sections of vein grafts was performed for histological examination.

### Flow cytometry

For characterization of BMSCs, BMSCs were analysed by fluorescence‐activated cell sorting (FACS). Cells in the fourth passage were incubated in antimouse/rat CD29 FITC (1:200, eBioscience), anti‐rat CD44H PE (1:300, eBioscience), anti‐rat CD45 APC (1:400, eBioscience), antimouse/rat CD90.1 PerCP‐cyanine5.5 (1:400, eBioscience) or CD34 antibody (ICO115) FITC (1:10 dilution, Santa Cruz) at concentrations specified by the manufacturer. Corresponding isotype identical antibodies served as controls, and the concentrations of the isotype identical antibodies were the same as the labelled antibodies.

Regarding CXCR4 expression, cells were stained with anti‐CXCR4 antibody (1:50, Abcam) or IgG isotype control, and the secondary antibody was F (ab’) 2 donkey anti‐rabbit IgG PE (1:50, eBioscience). To study the effects of simvastatin on the surface expression of CXCR4, 1 μmol/l simvastatin was added into the culture medium 48 h before harvest. To determine the effect of miR‐9, cells were harvested 48 h after transient transfection.

### RNA interference assay

Lipofectamine‐2000 transfection reagent (Invitrogen) was used for the transient transfection of BMSCs according to the manufacturer's instructions. Cells were transfected with 80 nM of miR‐9 mimic, miR‐9 inhibitor or their respective scrambled controls (Gene Pharma, Suzhou, Jiangsu, China) when they reached 70–80% confluence. Lipofectamine‐2000‐RNA complexes were allowed to form for 20 min at room temperature before addition to the cells.

### Migration assay

Chemotaxis experiments were performed with Costar ^®^ TranswellTM Permeable Supports (Corning) with membranes with 8‐μm pores. The cells were adjusted to a density of 2 × 105 cells/ml, and then 150 μL of cells were seeded in the upper chamber. After 12 h, the insert was removed, and non‐migrated BMSCs were scraped off the membrane with a rubber scraper. The cells that migrated to the lower surface of the membrane were then stained with crystal violet and counted in 5 randomly selected optical fields (×200).

Prior to the migration assay, the BMSCs were incubated with or without LY294002 (30 μmol/l) for 2 h prior to treatment with simvastatin (1 μmol/l) for 48 h or an SDF‐1α/CXCR4 cascade antagonist, AMD 3100 (5 μg/ml), for 1 h prior to treatment with simvastatin (1 μmol/l) for 48 h. Transient transfection of the inhibitor occurred for 48 h before the cells were collected for the migration assay.

### Western blot

CXCR4, p‐AKT and AKT expression was analysed by Western blot. A total of 30 μg protein extract was loaded onto a polyacrylamide gel (10%) used for electrophoresis. Then, the protein was transferred onto a polyvinylidene fluoride (PVDF) membrane. The membrane was incubated in TBST containing 5% non‐fat dry milk for 1 h at room temperature. The membranes were then incubated with anti‐CXCR4 antibody (1:1000, Abcam), p‐AKT1/2/3 antibody (Ser 473) (1:1000, Santa Cruz), AKT1/2/3 antibody (H‐136) (1:1000, Santa Cruz) or rabbit anti‐GAPDH (1:1000, Good Here) at 4°C overnight followed by peroxidase‐conjugated AffiniPure goat anti‐rabbit IgG (H+L) (1:5000) for 1 h at room temperature. Immunoreactivity was revealed by chemiluminescence. The relative integrated density values were measured based on the GAPDH protein expression levels as the control.

Different concentrations of simvastatin were added into the culture medium at different concentrations 48 h before the Western blot analysis as follows: 0. 1 μmol/l, 0.33 μmol/l, or 1 μmol/l. To examine the effect of p‐AKT on CXCR4 expression, the BMSCs were pre‐treated with or without the PI3K inhibitor LY294002 (30 μmol/l) for 2 h prior to treatment with simvastatin (1 μmol/l) for 48 h.

### RNA extraction and real‐time quantitative polymerase chain reaction (PCR)

We focused on the microRNAs with a predicted target of CXCR4 with good miSVR scores according to miRanda (version 2010). For the assessment of these microRNAs, small molecule RNA was isolated using RNAiso for Small RNA (Takara) according to the manufacturer's instructions 24 h after simvastatin and LY294002 were added to the culture medium. A total of 1 μg small molecule RNA was reverse‐transcribed using the All‐in‐One^™^ miRNA First‐Strand cDNA Synthesis Kit (GeneCopia). Real‐time PCR was performed with the All‐in‐OneTM miRNA qPCR Kit. PCR conditions were according to the kit, and a 1/10 dilution of cDNA was used. The reactions were performed with a LightCycler 2.0 Instrument. Each run included negative controls, and 5sRNA was selected as the housekeeping gene for normalization. Expression levels were calculated using the ∆∆Ct method.

### Bioinformatics prediction and luciferase reporter assay

The common miR‐9 targets predicted by computer‐aided algorithms were obtained using the multiple target prediction programs, TargetScan 5.2, the online miRNA prediction tool of the PicTar, and miRanda. BMSCs were plated into 6‐well plates and cotransfected with Lipofectamine 2000 with 2 μg of the luciferase reporter plasmids together with 80 nmol/L of miR‐9, the miR‐9 mimic scrambled control or nothing. Forty‐eight hours after the cells were transfected, luciferase assays were performed with a luciferase assay kit (E1910, Promega Corporation, Madison, WI, USA) according to the manufacturer's instructions.

### Statistical analysis

All experiments were repeated at least three times. Statistical significance (*P* < 0.05) was determined using Student's *t*‐test and one‐way ANOVA of variance followed by Dunnett's multiple comparison tests. All analyses were performed with the SPSS19.0 software (Chicago, IL, USA).

## Results

### Characterization of MSCs in *in vitro* culture

**Figure 1:**
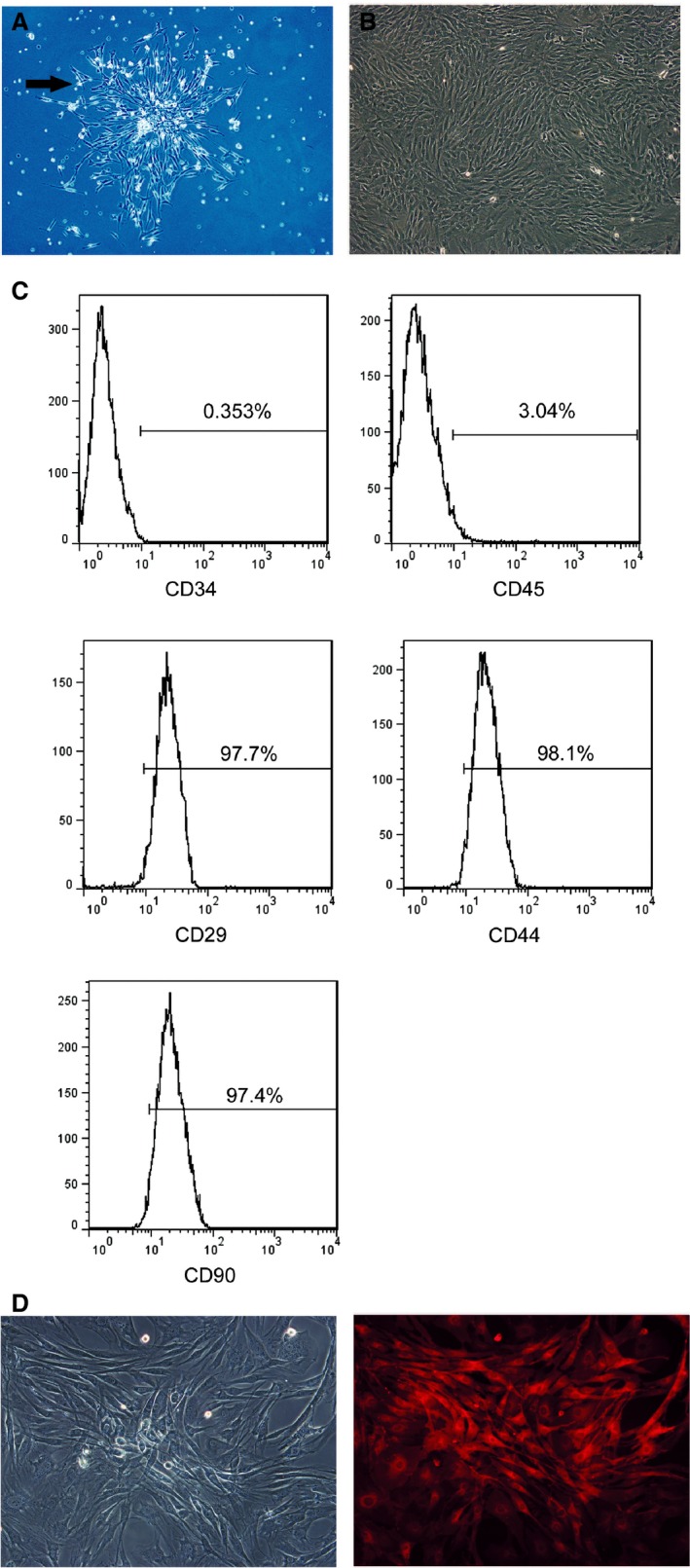
Characterization of BMSCs. (**A**) BMSCs exhibited a fibroblast–like shape and forms a cell colony (72 h after isolation, ×40. And the arrow indicates the cell colony). (**B**) Undifferentiated BMSCs after three passages of culture, display a flattened fibroblast‐like morphology (**C**). Flow cytometric analysis of cultured MSCs with CD29, CD44, CD90, CD34 and CD45 antibodies. BMSCs used in our study express CD90 and CD44, showing their mesenchymal origin. (**D**) Labelled cells showed red fluorescence under excitation.

Cell colonies were formed in the culture dishes, and approximately all adherent cells exhibited typical fibroblast cell morphology *in vitro* at passage 3 of the initial culture and maintained a similar morphology throughout passaging (Fig. 1A and B). The analysis of the cell surface antigens of early‐passage BMSCs (up to passage 5) was performed by flow cytometry. Cells of interest were defined as CD29^+^/CD34^−^/CD44^+^/CD45^−^/CD90^+^ (Fig. 1C). Almost all the cells exposed to CM‐DiI showed red fluorescence under fluorescence microscope. (Fig. 1D)

### Simvastatin promotes CXCR4 expression

**Figure 2:**
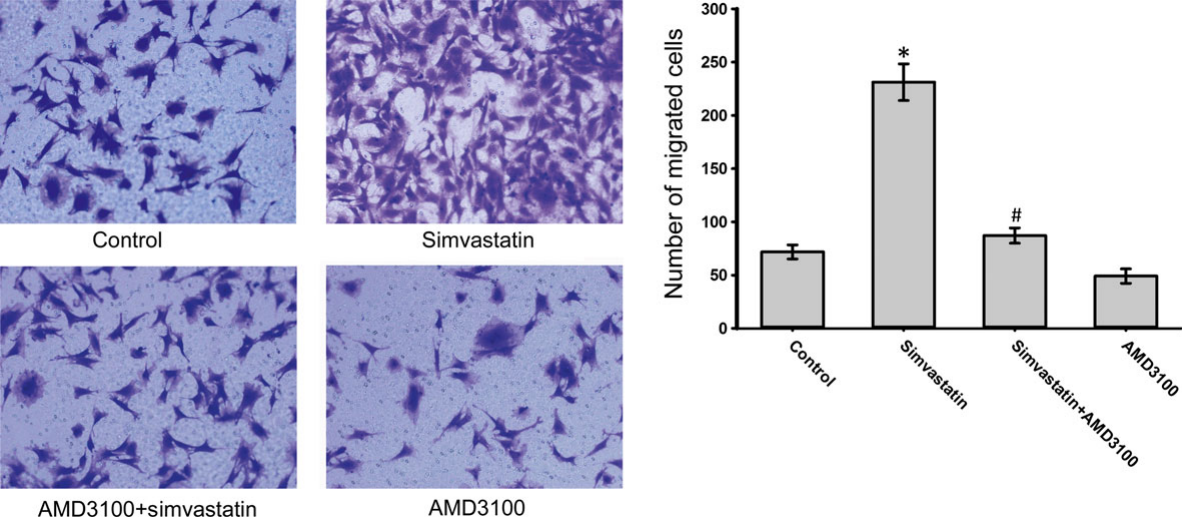
Simvastatin improves SDF‐1α‐induced migration of BMSCs. (**A**) Untreated BMSCs (Control), BMSCs treated with simvastatin (Simvastatin), and BMSCs pre‐treated with AMD3100 prior to simvastatin (AMD3100 + simvastatin) were placed in the upper chamber. The migrated cells were observed under a microscope (×200) *P < 0.05, *versus* Control group; ^#^ *P* < 0.05, compared with the Simvastatin group.

**Figure 3:**
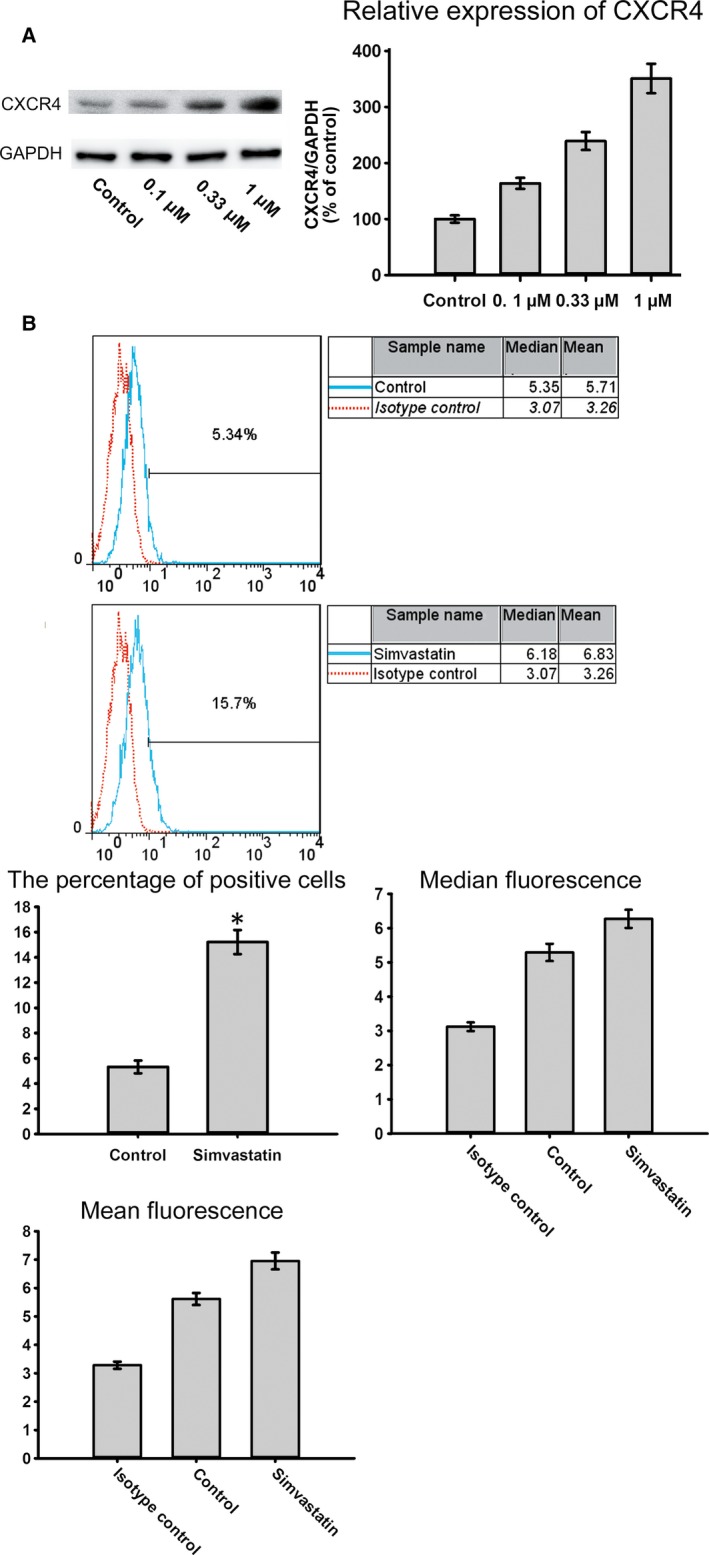
(**A**) The expression level of CXCR4 in BMSCs under the effect of different concentrations of simvastatin. The ratio of the expression of CXCR4 in different groups to that of the control group was calculated. (**B**) The surface expression of CXCR4 in the BMSCs increased following treatment with simvastatin.

**Figure 4:**
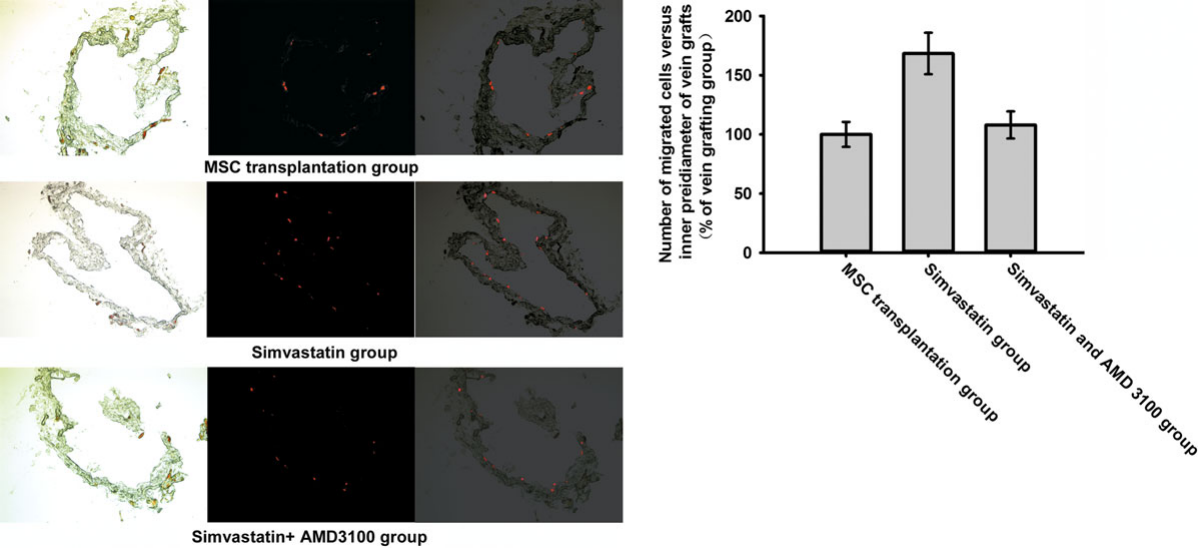
Morphology of frozen sections of each group was showed in the left picture. The fluorescence of labelled cells was showed in the middle one and the right one was formed by merging the first two pictures. Labelled BMSCs were more in simvastatin group in vein grafts with similar inner perimeter than MSC transplantation group and simvastatin and AMD 3100 group.

**Figure 5:**
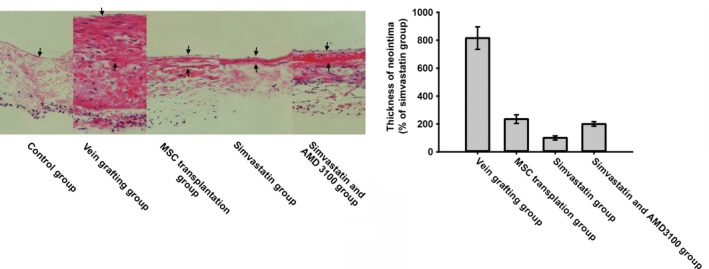
HE staining sections of vein grafts from week 4 after operation were analysed for neointima formation. The arrows indicate the control vessel wall in control group and neointima in the rest groups. Formation of neointima of vein grafts was most reduced in simvastatin group.

To evaluate the effect of simvastatin on BMSC homing, chemotaxis assays were performed. The data indicated that the exposure of BMSCs to simvastatin significantly increased SDF‐1α‐induced migration. Moreover, AMD 3100 was used to confirm that this increase was mediated by CXCR4. Pre‐treatment with AMD 3100 significantly reduced this increase (Fig. 2). CXCR4 expression under different concentrations of simvastatin was measured by Western blotting. Simvastatin caused a dose‐dependent increase in CXCR4, which reached a maximum at 1 μmol/l of simvastatin (Fig. 3A). Cell surface expression was measured by flow cytometry analysis. The percentage of CXCR4‐positive cells was significantly increased after treatment with simvastatin (Fig. 3B). Homing of BMSCs was examined by vein grafting model. We employed the number of CM‐DiI labelled cells *versus* the inner perimeter of vein grafts as the migration index of BMSCs. Labelled BMSCs migrated to the vein graft significantly increased in simvastatin group and this increase was brought down by the chemokine receptor antagonist for CXCR4, AMD 3100. (Fig. 4) Neointima of simvastatin group was also thinner than the MSC transplantation group and this trend was also partly reversed by AMD 3100. (Fig. 5) These data demonstrated that CXCR4 expression in BMSCs was up‐regulated by simvastatin treatment.

### Simvastatin promotes CXCR4 expression *via* the PI3K/AKT pathway

**Figure 6:**
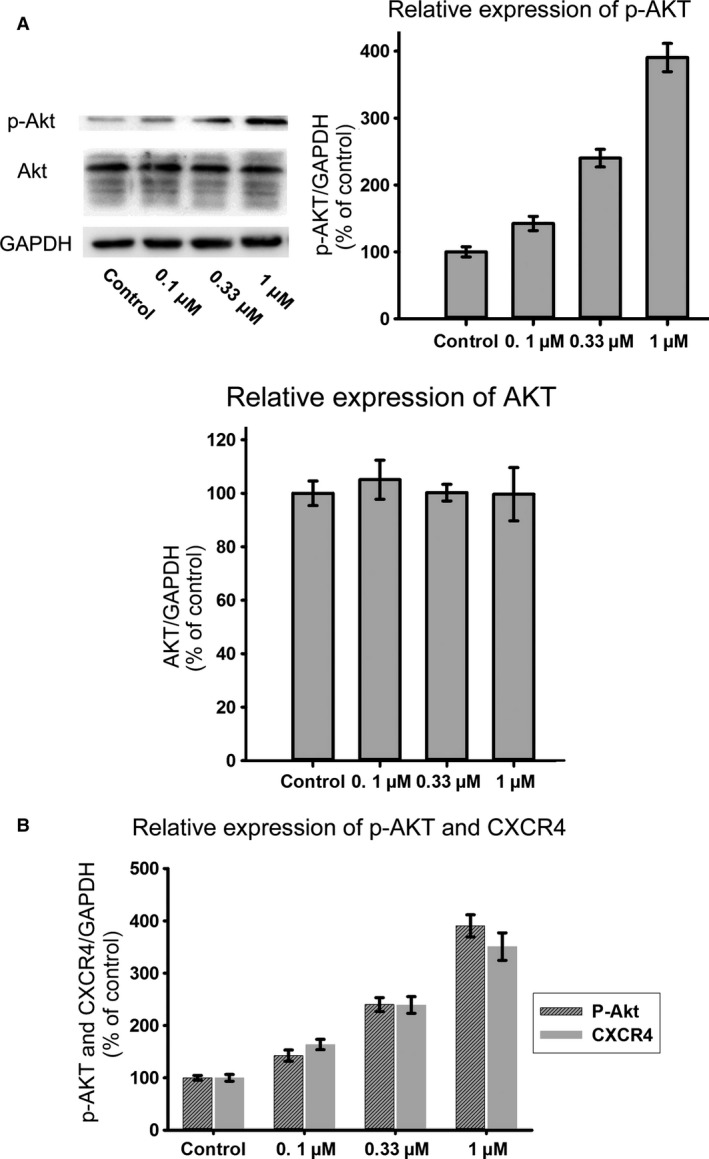
(**A**) Different concentrations of simvastatin led to different expression levels of p‐AKT in BMSCs; however expression of AKT showed no significant change. (**B**) As the concentration of simvastatin increased, expression of p‐AKT and CXCR4 increased synchronously.

**Figure 7:**
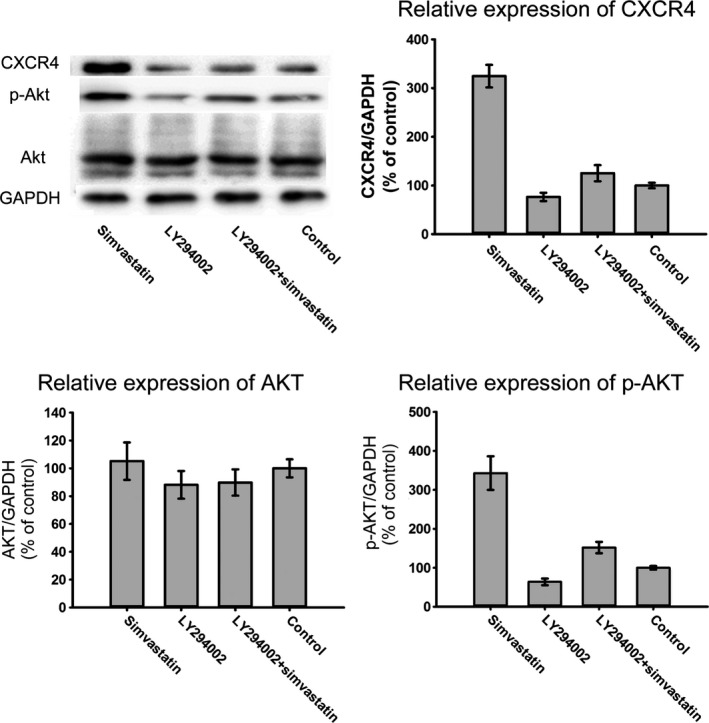
Western blot analysis of the protein expression levels of CXCR4, p‑AKT and AKT in BMSCs treated with simvastatin and/or LY294002. Treatment of LY294002 could partially inhibit phosphorylation of AKT and expression of CXCR4.

**Figure 8:**
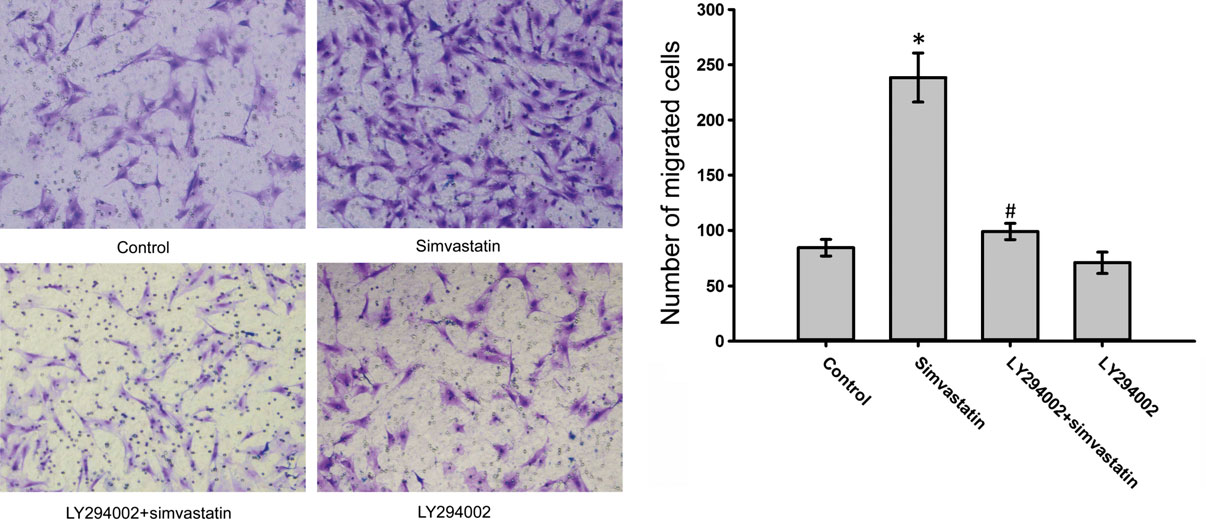
Effects of simvastatin and/or LY294002 on the migration of BMSCs to SDF‑1α. LY294002 could reduce the improvement of migration caused by simvastatin. The migrated cells were observed under a microscope (×200). *P < 0.05, *versus* Control group; ^#^ *P* < 0.05, compared with the Simvastatin group.

To determine whether simvastatin promoted the phosphorylation of AKT in BMSCs, p‐AKT expression levels were examined following treatment with simvastatin. Simvastatin treatment increased p‐AKT expression in a dose‐dependent manner, whereas the AKT protein levels were not significantly altered (Fig. 6A). CXCR4 expression may be related to p‐AKT expression because as the phosphorylation of AKT increased, the expression of CXCR4 increased (Fig. 6B). To confirm this hypothesis, we used LY294002. Treatment with LY294002 abolished the overexpression of CXCR4 caused by simvastatin (Fig. 7). Moreover, the chemotaxis assay results also supported this finding (Fig. 8). These results confirmed that simvastatin induced the up‐regulation of CXCR4 *via* the PI3K/AKT pathway.

### Simvastatin inhibits miR‐9 expression

To confirm the participation of miRs in the overexpression of CXCR4 caused by simvastatin, the expression of 40 miRs, which were predicted to target CXCR4 based on good mirSVR scores, was examined. As shown in Table 1, miR‐21, miR‐369‐3p, miR‐132 and miR‐9 expression was down‐regulated by simvastatin in BMSCs, and miR‐9 was the most down‐regulated miR.

**Table 1:** Expression of miRs predicted to target CXCR4 under the effect of simvastatin

miRNA	Mean	SD	miRNA	Mean	SD
rno‐miR‐9	0.17	0.01	rno‐miR‐223	2.42	0.57
rno‐miR‐21	0.35	0.08	rno‐miR‐125b‐2‐3p	2.60	0.84
rno‐miR‐369‐3p	0.64	0.15	rno‐miR‐326	2.64	1.41
rno‐miR‐132	0.69	0.16	rno‐miR‐338‐5p	2.83	0.83
rno‐miR‐381	0.80	0.12	rno‐miR‐338	2.83	1.21
rno‐miR‐330	1.24	0.06	rno‐miR‐7a‐1‐3p	2.93	1.95
rno‐miR‐342‐3p	1.26	0.35	rno‐miR‐532‐5p	3.01	1.16
rno‐miR‐540	1.61	0.73	rno‐miR‐151‐3p	3.44	2.28
rno‐miR‐290	1.67	0.36	rno‐miR‐139‐5p	4.24	1.28
rno‐miR‐873	1.70	0.51	rno‐miR‐410	4.57	1.33
rno‐miR‐484	1.80	0.36	rno‐miR‐340‐5p	4.79	1.75
rno‐miR‐674‐3p	1.82	0.21	rno‐miR‐212	6.00	1.73
rno‐miR‐291‐a‐3p	1.90	0.71	rno‐miR‐336	8.12	3.33
rno‐miR‐673	1.93	0.67	rno‐miR‐448	9.60	1.39
rno‐miR‐204‐3p	2.01	1.00	rno‐miR‐29b‐1‐5p	12.40	5.11
rno‐miR‐494	2.20	0.62	rno‐miR‐708‐3p	12.51	2.32
rno‐miR‐224	2.20	0.66	rno‐miR‐194	13.69	2.48
rno‐miR‐377	2.23	0.49	rno‐miR‐220	14.14	1.57
rno‐miR‐344‐5p	2.26	0.59	rno‐miR‐105	14.45	1.99
rno‐miR‐148b‐5p	2.34	0.11	rno‐miR‐879	17.98	4.49

Simvastatin led to different expression of miRNAs predicted to target CXCR4. Expression of miR‐9, miR‐21, miR‐369‐3p, miR‐132, and miR‐381 decreased after treatment of simvastatin. Other miRs expression increased in different extent. Among those miRNAs, miR‐9 had the largest descent.

### MiR‐9 targets CXCR4 and regulates BMSC migration

**Figure 9:**
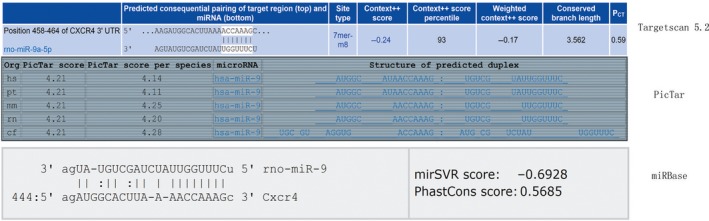
MiR‐9 might target CXCR4 with good scores according to TargetScan 5.2, the online miRNA prediction tool of the PicTar, and miRBase.

**Figure 10:**
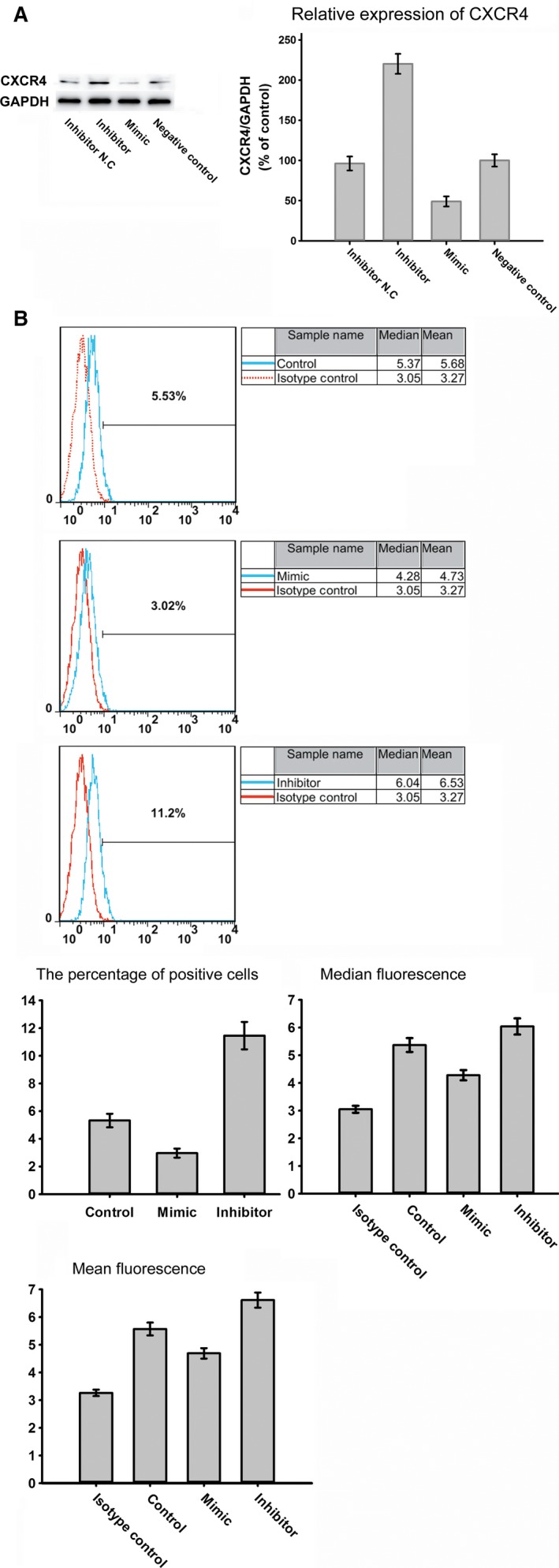
(**A**) The expression level of CXCR4 in BMSCs after transfection with inhibitor, Inhibitor N. C, mimic and negative control. (**B**) The surface expression of CXCR4 in the BMSCs following transfection.

**Figure 11:**
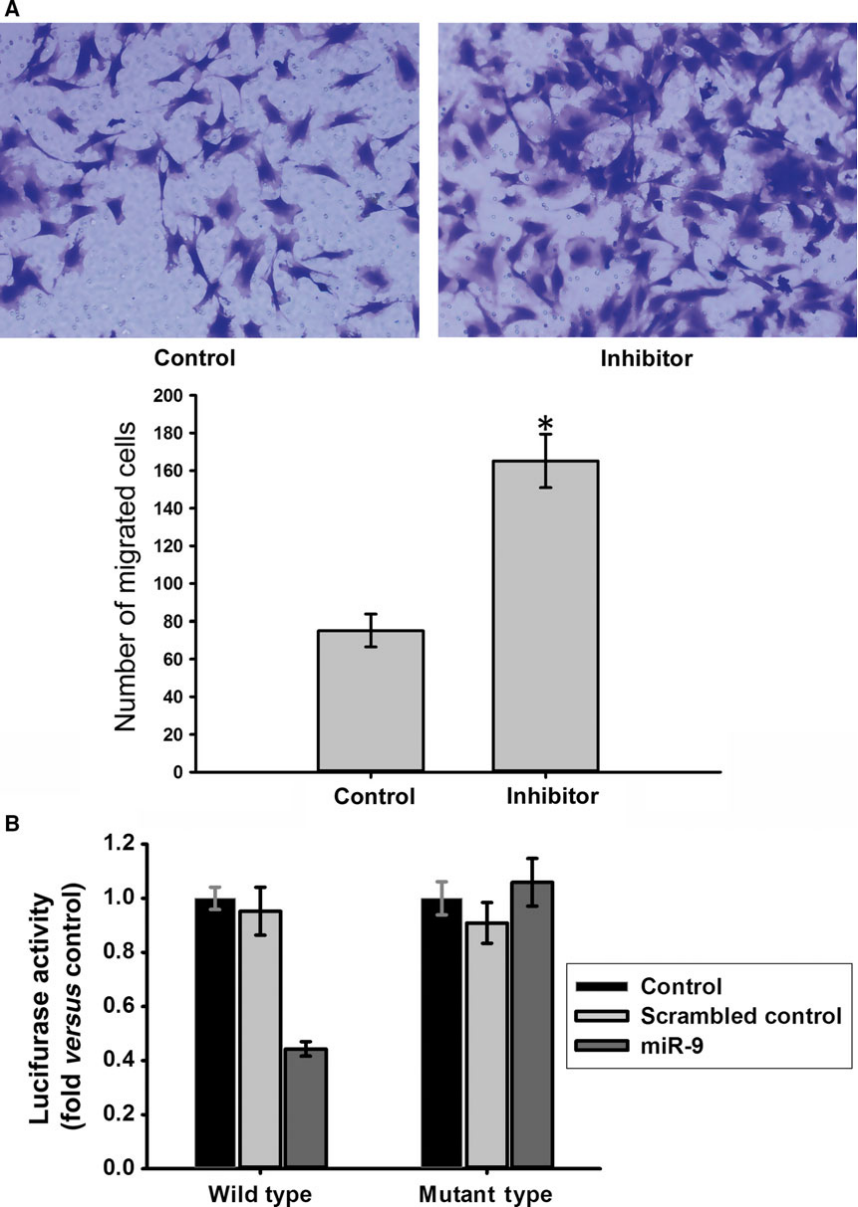
(**A**) Effects of miR‐9 on the migration of BMSCs to SDF‑1α. Transfection with inhibitor led to an up‐regulation of migration. *P < 0.05, *versus* Control group (**B**) Luciferase activity was suppressed by miR‐9, but not scrambled control in the wild‐type reporter group. In the mutant‐type reporter group, luciferase activity showed no significant difference under different treatment. The wild‐type reporter contains the full‐length 3′UTR of rat CXCR4 mRNA including the sequence complementary to miR‐9 and this sequence is mutated in mutant‐type reporter. *P < 0.05, *versus* Control group.

According to TargetScan 5.2, the online miRNA prediction tool PicTar, and miRanda, miR‐9 may target CXCR4 with good scores (Fig.9). Because miR‐9 expression was significantly down‐regulated in BMSCs treated with simvastatin and simvastatin promoted CXCR4 expression, we examined whether miR‐9 regulates CXCR4 expression. As shown in Fig. 10A, miR‐9 knockdown by a miR‐9 inhibitor markedly increased CXCR4 expression in BMSCs, whereas the miR‐9 mimic reduced CXCR4 expression. The inhibitor also increased the surface expression of CXCR4, and the miR‐9 mimic produced the opposite effect (Fig. 10B). To investigate the effect of miR‐9 on the homing of BMSCs, we performed a cell migration assay. Knockdown of miR‐9 significantly increased the number of migrated BMSCs (Fig. 11A).

To confirm whether CXCR4 is a target of miR‐9, a luciferase reporter assay was performed. The relative luciferase activity of the wild‐type reporter was reduced by the miR‐9 mimic. By contrast, the relative luciferase activity of the mutant‐type reporter was not significantly affected by the miR‐9 mimic compared with the scrambled control, indicating that miR‐9 may regulate CXCR4 expression by binding to the sequence in the 3′‐untranslated region (UTR) (Fig. 11B).

### The PI3K/AKT pathway may participate in the regulation of miR‐9

**Figure 12:**
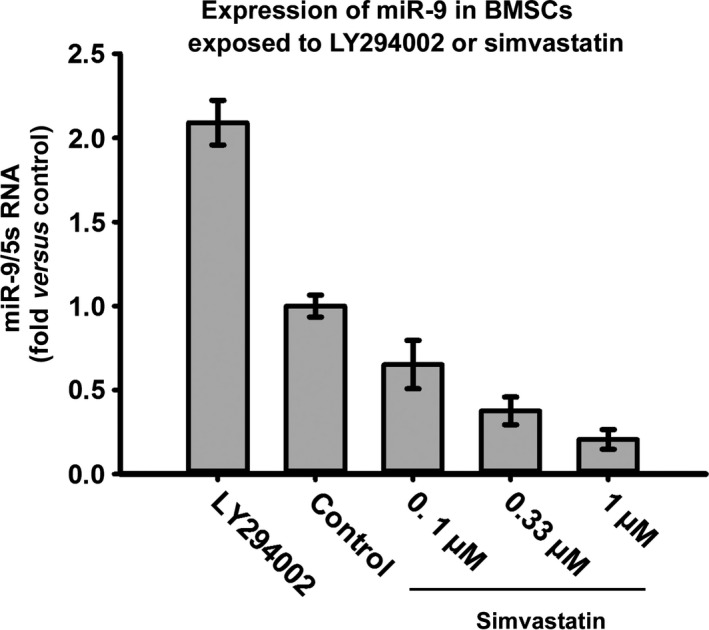
Expression of miR‐9 might be related to the phosphorylation of AKT. As the concentration of simvastatin increased, expression of mir‐9 decreased which was opposite to the trend of p‐AKT. LY294002 could increase expression of mir‐9.

Because simvastatin promotes the phosphorylation of AKT and down‐regulates the expression of miR‐9, we examined whether miR‐9 expression is related to the PI3K/AKT pathway. As the concentration of simvastatin increased, the expression of miR‐9 decreased. The expression of miR‐9 and p‐AKT exhibits an opposite trend, indicating that a relationship between the two may exist. Moreover, LY294002 increased the expression of miR‐9 (Fig. 12). Therefore, we predict that PI3K/AKT pathway activation may inhibit miR‐9 expression.

## Discussion

This study demonstrates that simvastatin promotes SDF‐1α‐induced BMSC migration, and this effect is mediated by the PI3K/AKT signalling pathway. MiR‐9 also participates in this process, and the expression of miR‐9 is affected by the phosphorylation of AKT. The promotion of the homing of BMSCs indicates that simvastatin may have beneficial effects beyond its cholesterol‐lowering properties in stem cell therapy.

BMSCs may be a promising treatment for numerous diseases, such as bone defects, immune‐mediated diseases, and acute myocardial infarction (AMI). However, their clinical application remains debated. Although PFKB3 and PFK may be used to distinguish cancer stem cells, the propensity to form teratomas of stem cell worries us 25, 26. Survival, division,differentiation and migration affect the therapeutic applications of stem cells. Stem cell niche and cell adhesion molecules provide support and maintenance of the stem cell and regulate its proliferation, differentiation migration, and so on 26, 27, 28. It is a very important problem that BMSCs home to tissues or organs to be repaired. The pathotropism of BMSCs may be similar to the chemotaxis of leucocytes during inflammation. Various chemokines, cytokines and integrins may be involved 29. The SDF‐1α/CXCR4 cascade is a central regulator of the recruitment of stem cells to tissues that must be repaired. SDF‐1α promotes CXCR4^+^ MSC egress from the stem cell niche, and these cells home to damaged sites along the gradient of SDF‐1α 30. For example, the enhanced expression of SDF‐1α after myocardial infarction results in the increased engraftment of BMSCs into the infarcted myocardium 31. At injury sites, tissue ischaemia induces SDF‐1α expression and enhances stem cell gathering 32. This cascade also participates in bone repair, and the inhibition of SDF‐1α or blocking of its receptor, CXCR4, prevents MSC recruitment and results in impaired bone healing 33. However, *in vitro* culture of BMSCs leads to the loss of surface expression of CXCR4 34. This phenomenon may lead to impairment of the therapeutic effects of BMSCs. To improve the clinical use of BMSCs, we must enhance CXCR4 expression in BMSCs. Several strategies are available to augment or stabilize the expression of CXCR4, including genetic modification by viruses or plasmids and the alteration of culture conditions. However, the high cost and adverse side effects of these strategies have limited their application 35. In our study, simvastatin promoted the migration of BMSCs to SDF‐1α, and this effect was reversed by AMD 3100. Moreover, the total and cell surface expression of CXCR4 in BMSCs was also enhanced. To find whether simvastatin could improve homing of CXCR4 *in vivo*, vein grafting model was established. As proved in previous studies, SDF‐1α is over‐expressed in vein grafts 36, 37, 38. So the CXCR4^+^ BMSCs would migrate to the vein grafts and the higher the CXCR4 expression the more the migrated cells. This was in accordance with our results: Simvastatin enhanced homing of BMSCs to vein graft in *via* up‐regulation of CXCR4 expression and AMD3100 could inhibit this enhancement. Transplantation of BMSCs can help to rebuild the endothelium in vein grafts, and then alleviate the formation of neointima 22. So simvastatin could improve this effect by increasing the number of migrated BMSCs to vein grafts. It did do this in our experiments as showed in Fig 5. This finding is consistent with a previous report that demonstrates that simvastatin significantly increases CXCR4 expression in BMSCs and increases BMSC migration to rat brain microvascular endothelial cells (RBMECs) and astrocytes 23. In our opinion, the overexpression of CXCR4 may play an important role in the increased numbers of DAPI‐labelled cells in the hearts of pigs treated with SIMV + BMSCs compared with pigs exclusively treated with BMSCs in the study by Yang, because of the increased migration of BMSCs. Simvastatin is safe and inexpensive, so the combination of simvastatin and BMSCs may have great clinical benefits.

The PI3K/AKT signalling pathway is an important mediator of cell biology. Nuclear localized AKT affects cell cycle, stemness, survival, proliferation and clonogenicity 39. Simvastatin enhances the phosphorylation of AKT in numerous cell types, such as EPCs and endothelial cells 13, 14. Incubation of THP‐1 cells with simvastatin induces the phosphorylation of AKT and regulates IL‐1β secretion 40. Inhibition of RhoA by simvastatin induces increased AKT phosphorylation in vascular smooth muscle cells 41. The neuroprotective effects of simvastatin are mediated by p‐AKT. Treatment with LY294002 inhibits these effects in C5a‐induced neurotoxicity in cortical cells 42. However, research indicates that simvastatin also inhibits AKT phosphorylation. Simvastatin pre‐treatment attenuates TNF‐α‐elicited AKT phosphorylation in cultured aortic smooth muscle cells 43. Simvastatin leads to impaired AKT phosphorylation and results in reduced protein synthesis in C2C12 myotubes 44. Simvastatin has complex effects on the PI3K/AKT signalling pathway in different cell types, and we determine whether simvastatin activates AKT in BMSCs. We found that AKT phosphorylation increased with increasing concentrations of simvastatin. Simvastatin affects the biological features of BMSCs *via* the PI3K/AKT signalling pathway.

The PI3K/AKT signalling pathway affects the expression of CXCR4 in stem cells. This pathway participates in hypoxia preconditioning‐induced HIF‐1ɑ expression and up‐regulates CXCR4 expression in BMSCs 45. BMSC pre‐treatment with LY294002 also suppresses the COMP‐Ang1‐stimulated migration of cells. Exendin‐4 augments the SDF‐1α/CXCR4 cascade by activating this pathway in adipose‐derived stem cells, and LY294002 treatment significantly inhibits this effect 46. The PI3K/AKT signalling pathway also enhances CXCR4 expression in various types of tumour cells, such as hepatoma cells 47, colorectal cancer cells 48, and prostate tumour cells 49. Because simvastatin‐activated AKT in BMSCs and enhanced the expression of CXCR4 in BMSCs, the question of whether p‐AKT participated in the simvastatin‐induced expression of CXCR4 in BMSCs was posed. CXCR4 expression in BMSCs exhibited a trend that is similar to that of p‐AKT in response to different concentrations of simvastatin. In control groups with low levels of p‐AKT, CXCR4 expression was minimal, and the highest phosphorylation of AKT appeared in the 1 μmol/l simvastatin group with the maximal expression of CXCR4. Moreover, this trend was inhibited by LY294002, suggesting that the PI3K/AKT signalling pathway participates in simvastatin‐induced CXCR4 expression. LY294002 also reversed the enhanced number of BMSCs migrating to SDF‐1α caused by simvastatin. These results reveal that the PI3K/AKT pathway is essential for the simvastatin‐induced homing of BMSCs, similar to COMP‐angiopoietin 1, hypoxia and Exendin‐4.

MiRs participate in almost all biological features of cells, and some miRs play a direct role in the regulation of CXCR4. For example, miR‐494 suppresses cell proliferation and the invasion of breast cancer cells by targeting CXCR4 50. The up‐regulation of miR‐146a decreases CXCR4 expression in a leukaemic monocytic cell line and CD4 (+) T lymphocytes 51. The decreased expression of miR‐9 leads to the constitutive activation of β‐catenin *via* the overexpression of CXCR4 in oral squamous cell carcinoma (OSCC) cells 52. miR‐150 regulates CXCR4 expression in mouse bone marrow‐derived mononuclear cells 18. Several miRs may participate in the regulation of CXCR4 in rat BMSCs caused by simvastatin. We found that simvastatin down‐regulated miR‐21, miR‐369‐3p, miR‐132 and miR‐9 expression in rat BMSCs. MiR‐9 targeted CXCR4 in rat BMSCs. Transfection of a miR‐9 inhibitor markedly increased CXCR4 expression in BMSCs, whereas a miR‐9 mimic reduced CXCR4 expression. Transfection of a miR‐9 inhibitor also improved the surface expression of CXCR4 and enhanced the number of cells migrating to the bottom of the transwell membrane.

Both p‐AKT and miR‐9 regulate the expression of CXCR4 in BMSCs. These proteins may be related; therefore, we determined the expression of miR‐9 in BMSCs after treatment with LY294002 and different concentrations of simvastatin. MiR‐9 expression exhibited an opposite trend compared with p‐AKT upon exposure to different concentrations of simvastatin, and LY294002 improved miR‐9 expression. These data suggest that p‐AKT regulates miR‐9 expression.

In this study, we demonstrate that simvastatin increases the expression of CXCR4 and SDF‐1α‐induced migration in BMSCs *via* the PI3K/AKT/miR‐9 pathway. These data may lead to the development of a new and safe method for the application of BMSCs for many diseases, although many aspects of this must be studied. Because simvastatin activates the PI3K/AKT pathway, it may affect the proliferation and survival of BMSCs. Furthermore, the mechanism by which PI3K/AKT affects miR‐9 expression in BMSCs must be elucidated as well as whether this mechanism occurs in other cells. We must also determine whether inhibition of miR‐9 is safe, which will be the focus of continuing research efforts.

## Conflict of interest

The authors confirm that there are no conflicts of interest.
